# Duodenal perforation: an unusual complication of sickle cell anemia

**DOI:** 10.11604/pamj.2014.18.217.4645

**Published:** 2014-07-16

**Authors:** Can Acıpayam, Güliz Aldıç, Bülent Akçora, Mehmet Emin Çelikkaya, Hasan Aşkar, Bayram Ali Dorum

**Affiliations:** 1 Mustafa Kemal University, School of Medicine, Department of Pediatric Hematology and Oncology, Hatay, Turkey; 2 Mustafa Kemal University, School of Medicine, Department of Pediatrics, Hatay, Turkey; 3 Mustafa Kemal University, School of Medicine, Department of Pediatric Surgery, Hatay, Turkey

**Keywords:** Sickle cell anemia, duodenal perforation, abdominal pain

## Abstract

Duodenal perforation in childhood is a rare condition with a high mortality rate if not treated surgically. Primary gastroduodenal perforation is frequently associated with peptic ulcer and exhibits a positive family history. Helicobacter pylorus is the most significant agent. Secondary gastroduodenal perforation may be a finding of specific diseases, such as Crohn disease, or more rarely may be associated with diseases such as cystic fibrosis or sickle cell anemia. A 14-year-old boy presented with abdominal and back pain. The patient was operated on for acute abdomen and diagnosed with duodenal perforation. Helicobacter pylorus was negative. There was no risk factor to account for duodenal perforation other than sickle cell anemia. Surgical intervention was successful and without significant sequelae. Duodenal perforation is a rare entity described in patients with sickle cell anemia. To our knowledge, this is the first report of duodenal perforation in a patient sickle cell anemia.

## Introduction

Sickle cell disease is an inherited condition resulting from a point mutation occurring in the triplet codon of the gene for the B globin chain of hemoglobin. Abdominal pain is a frequent symptom among patients undergoing sickle cell crisis. The abdominal symptoms that may be comorbid with sickle cell crisis are generally attributed to circulatory stasis and vascular occlusion. Sickle hemoglobinopathy-related gastrointestinal disorders include cholecystitis, abdominal pain crises (potentially associated with ischemic bowel injury), paralytic ileus, splenic sequestration and acute hepatic syndromes [1, 2].

Peptic ulcer disease is the main cause of gastroduodenal perforation, followed, in order, by necrotic or ulcerated malignancies, iatrogenic injuries and traumatic injuries [3–5]. Surgery represents the essential element of treatment for gastroduodenal perforation. Gastroduodenal perforation associated with peptic ulcers frequently occurs in the gastric antrum and the duodenal bulb. The most frequent and regular finding in gastroduodenal perforation is extraluminal free air. However, this may not be present, especially at the onset of symptoms [5]. Although there have been previous reports of ischemic bowel injury in the gastrointestinal system in patients with sickle cell anemia, we encountered no cases of gastroduodenal perforation [6]. Duodenal perforation is a rare finding in patients with sickle cell anemia. We report a case of a 14-year-old child with duodenal perforation associated with sickle cell anemia.

## Patient and observation

A 14-year-old boy with homozygous sickle cell disease presented to hospital with abdominal pain, anorexia and intermittent vomiting over the previous 4 days. The pain was severe. His medical history was significant for sickle cell anemia. He had been diagnosed with sickle cell anemia at the age of 3 and had not received regular follow-ups. The most recent painful crisis had taken place 4 years previously and he had experienced no gastric symptoms to date. He was not using hydroxyurea.

At physical examination, the patient′s temperature was 36.7 °C, heart rate was 96 beats/min, respiratory rate 20/min and blood pressure 100/60 mmHg. Examinations of the head, ears, eyes, nose and throat were within normal limits. Respiration was normal, and cardiac examination detected no abnormalities. No organomegaly was present. We identified diffuse abdominal sensitivity to palpation and rebound tenderness.

**Figure 1:**
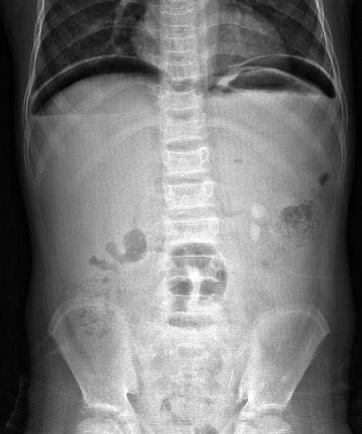
Free air beneath the diaphragm at abdominal x-ray film

**Figure 2:**
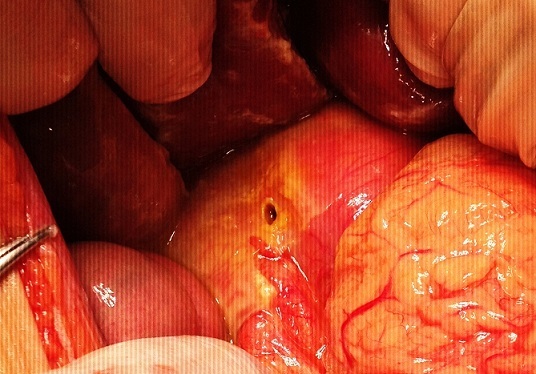
2x1 cm perforation in the anterior face of the second part of the duodenum in a 14-year-old child with sickle cell anemia

Hematological values were white blood count 66.800/mm3, hemoglobin 7.8 g/dl and platelets 1.099.000/mm3. Coagulation profile and biochemical parameters were normal. Helicobacter pylori IgG and IgM values were negative. Abdominal x-ray film revealed free air beneath the diaphragm (Figure 1). Surgical consultation was performed. Preoperative diagnosis was perforation. Manual exchange blood transfusion was performed to reduce HbS. HbS decreased to 26% and hemoglobin increased to 9.1 gr/dl after transfusion. A 2x1 cm perforation in the anterior face of the second part of the duodenum was detected at exploratory laparotomy, and primary repair was performed (Figure 2). No problem was encountered at postoperative observation, and the patient was discharged without sequelae.

## Discussion

Abdominal pain frequently accompanies sickle cell pain crisis, in which vaso-occlusive or infarctive crisis often leads to acute abdominal pain associated with ischemia and infarction. Approximately 10% of patients with sickle cell anemia are admitted to hospital each year due to acute abdominal pain. Factors implicated in this pain include vertebral marrow hyperplasia or infarct with nerve root compression, mesenteric and retroperitoneal lymphadenitis with infarction, and occlusion of blood supply to ischemic or infracted abdominal organs. Polymerization of HbS under low oxygen tension leads to distorted erythrocytes with reduced deformability. This in turn results in vascular occlusion and injury. The highly complex interaction between the endothelium, plasma factors, leucocytes, and rigid sickle red cells is also a significant factor in vaso-occlusion [6].

Abdominal pain is frequently observed during vaso-occlusive or infarctive crisis and may lead to acute surgical abdomen [7, 8]. Pre-operative transfusions are recommended for patients with sickle cell crisis undergoing surgical procedures under general anesthesia [8]. Preoperative transfusion was performed in our case, and HbS was brought down 26%. Mucosal ischemia associated with the microvascular abnormalities inherent in the condition may be responsible for the increased incidence of peptic ulcer disease in sickle cell anemia [2]. The most common cause of duodenal perforation is peptic ulcer disease [5]. One study reported peptic ulcer disease in 7.7% to 35% of patients with sickle cell anemia [1]. Lee et al. [9] investigated upper endoscopic and gastric acid output in 51 patients with homozygous sickle cell disease and recurrent epigastric pain. They determined 39% abnormalities in the upper gastrointestinal tract, including 18 (35%) cases with peptic ulcers [9]. Our patient presented with abdominal pain and was diagnosed with perforation after examinations. In contrast to the literature, he had no symptoms suggestive of peptic ulcer disease. No etiological factor other than sickle cell disease was detected. Duodenal perforation was attributed to mucosal ischemia and hypoxia resulting from 4-day vaso-occlusive crisis.

## Conclusion

Bearing gastroduodenal perforation in mind may be life-saving in patients with sickle cell anemia presenting with abdominal and back pain, even in the absence of any previous gastric symptoms.
